# Early prediction of therapy responses and outcomes in breast cancer patients using quantitative ultrasound spectral texture

**DOI:** 10.18632/oncotarget.1950

**Published:** 2014-05-07

**Authors:** Ali Sadeghi-Naini, Lakshmanan Sannachi, Kathleen Pritchard, Maureen Trudeau, Sonal Gandhi, Frances C. Wright, Judit Zubovits, Martin J. Yaffe, Michael C. Kolios, Gregory J. Czarnota

**Affiliations:** ^1^ Physical Sciences, Sunnybrook Research Institute, Sunnybrook Health Sciences Centre, Toronto, ON, Canada; ^2^ Department of Radiation Oncology, Odette Cancer Centre, Sunnybrook Health Sciences Centre, Toronto, ON, Canada; ^3^ Department of Medical Biophysics, Faculty of Medicine, University of Toronto, Toronto, ON, Canada; ^4^ Department of Radiation Oncology, Faculty of Medicine, University of Toronto, Toronto, ON, Canada; ^5^ Division of Medical Oncology, Sunnybrook Health Sciences Centre, University of Toronto, Toronto, ON, Canada; ^6^ Division of General Surgery, Sunnybrook Health Sciences Centre, Toronto, ON, Canada; ^7^ Department of Surgery, Faculty of Medicine, University of Toronto, Toronto, ON, Canada; ^8^ Department of Pathology, Sunnybrook Health Sciences Centre, University of Toronto, Toronto, ON, Canada; ^9^ Department of Physics, Ryerson University, Toronto, ON, Canada

**Keywords:** Personalized medicine, Therapy response monitoring, Breast Cancer, Neo-adjuvant chemotherapy, Tumor response heterogeneity, Textural analysis, Quantitative ultrasound

## Abstract

Early alterations in textural characteristics of quantitative ultrasound spectral parametric maps, in conjunction with changes in their mean values, are demonstrated here, for the first time, to be capable of predicting ultimate clinical/pathologic responses of breast cancer patients to chemotherapy. Mechanisms of cell death, induced by chemotherapy within tumor, introduce morphological alterations in cancerous cells, resulting in measurable changes in tissue echogenicity. We have demonstrated that the development of such changes is reflected in early alterations in textural characteristics of quantitative ultrasound spectral parametric maps, followed by consequent changes in their mean values. The spectral/textural biomarkers derived on this basis have been demonstrated as non-invasive surrogates of breast cancer chemotherapy response. Particularly, spectral biomarkers sensitive to the size and concentration of acoustic scatterers could predict treatment response of patients with up to 80% of sensitivity and specificity (p=0.050), after one week within 3-4 months of chemotherapy. However, textural biomarkers characterizing heterogeneities in distribution of acoustic scatterers, could differentiate between treatment responding and non-responding patients with up to 100% sensitivity and 93% specificity (p=0.002). Such early prediction permits offering effective alternatives to standard treatment, or switching to a salvage therapy, for refractory patients.

## INTRODUCTION

Patient response to cancer treatment is an important therapeutic parameter that is dependent on many factors including pathologic subtype, tumor grade, and stage of disease, as well as patient age, genetic-profile, and immune response to therapy, in addition to other factors. Therefore, a predefined therapy regimen does not result in an equivalent response in different patients, nor is it an effective treatment for all patients. This highlights the importance of early predictions of ultimate patient responses to cancer therapies. Detecting refractory response of a specific patient to a routine therapy at early stages can facilitate an alteration in primary treatment, or even a switch to an early salvage therapy, leading to potentially better outcomes [1,2]. This has in part motivated research towards the concept of personalized cancer therapy with much attention recently in many aspects of biomedical sciences [3-7].

Breast cancer patients represent a patient population who may benefit from changing ineffective cancer therapies to more efficacious treatments [8]. Breast cancer is a most frequently diagnosed cancer and the second cause of cancer related death in women [9]. Despite recent improvements with the detection of breast cancer at early stages, a considerable fraction of this patient population is diagnosed with late stage disease. An estimated 5-20% of newly diagnosed cases remain still classified as locally advanced breast cancer (LABC) [10,11], with even larger proportions outside of North America. Locally advanced breast cancer, according to clinical guidelines, includes stage III and a subset of stage IIB (T3N0) disease and often presents as tumors which are frequently greater than 5 cm, involving the chest wall and/or classified as inflammatory breast cancer. Locally advanced breast cancer patients generally have poor long-term survival rates (five-year survival rate of 55%, approximately) in comparison to the early stage patients, mainly due to the progression of the disease and a high risk of metastatic spread [11].

Locally advanced breast cancer patients are currently treated with aggressive therapeutic combinations, often with neo-adjuvant chemotherapy followed by surgery, radiation therapy, and when indicated, Herceptin and/or hormonal manipulation [12-14]. Within this therapeutic scenario, the importance of clinical and pathologic complete response to neo-adjuvant chemotherapy has been highlighted, in several studies, as a marker of better outcomes (with survival rates reaching 70% with complete response) [15,16]. However this prognostic factor is often assessed at the time of surgery, far too late to make any modification to a neo-adjuvant treatment. This is mainly due to the fact that standard clinical surrogates based on on-going physical assessment by palpation, or using conventional clinical imaging such as x-ray mammography, or B-mode ultrasound, suffer from an inability to objectively evaluate treatment response early on during a course of treatment [17].

Despite the complicated nature of responses to cancer treatments, tumor cell death frequently results in micro-structural and gross functional alterations in tumors which are measurable even at early stages [18-24]. These alterations are introduced through different physiological mechanisms and can be monitored using functional imaging techniques [25,26]. Accumulation of such early micro-alterations, in long term, results in macroscopic changes in the physical properties of tumors such as their size, which is currently used as a standard clinical criterion of tumor response to treatment [27,28]. Therefore, the quantification of these microscopic changes in tumor physiology has a high potential for accurately predicting ultimate tumor response early on during a course of treatment. In this context, a number of imaging modalities, including positron emission tomography (PET) [4], magnetic resonance imaging (MRI) [29,30], diffuse optical spectroscopy (DOS) [31,32], and elastography [33], have recently been demonstrated for evaluating cancer treatment responses within weeks to months after the start of treatment.

Investigators have recently undertaken genetic approaches for therapy response monitoring. Analyses of circulating tumor DNA to monitor breast cancer response to treatment early on during a course of treatment have shown promise [34]. However, regardless of providing useful scientific perspective, the proposed method is by necessity invasive requiring time-consuming analyses for quantification of circulating tumor DNA and gene sequencing. In terms of imaging modalities, PET remains costly, requiring radionuclide contrast agent, and thus limiting the number of times each patient can be evaluated during a treatment course. Although DOS and elastography have also shown potential for distinguishing treatment resistive patients, they remain investigational.

Clinical ultrasound (US) is a low-cost and portable imaging modality with a short imaging time and a relatively high spatial resolution. In recent preclinical studies quantitative ultrasound (QUS) techniques at both high and conventional-frequencies have been demonstrated in the detection and quantification of cell death in response to cancer treatments [23,35,36]. These include treatments with chemotherapy, photodynamic therapy, radiation therapy, anti-vascular treatment, or combined therapies [37]. A very recent pilot clinical study also investigated these techniques for evaluating patient responses to chemotherapy [38]. In particular, mid-band fit (MBF) and 0-MHz intercept spectral parameters which can be linked to ultrasound backscatter power, and size and concentration of acoustic scatterers [39,40], have shown promise. In addition, textural properties of QUS spectral parametric maps, including contrast, correlation, and homogeneity of MBF and 0-MHz intercept, have been investigated preclinically [41,42]. Such parameters that quantify the spatial relationship between neighboring acoustic scatterers within tissue micro-structures, have been demonstrated capable of characterizing response heterogeneities, with more sensitivity and higher levels of correlation to histological cell death, compared to mean values of the spectral parameters [41,42]. These parameters can be potentially applied for assessing response to neo-adjuvant chemotherapy in LABC patients.

Based on the rationale that responses in tumors are spatially inhomogeneous [43], this study evaluates, for the first time, the efficacy of textural characteristics of QUS spectral parametric maps for the prediction of tumor response to neo-adjuvant chemotherapy in LABC patients, early on during a course of treatment. Obtained results demonstrated statistically significant differences in changes observed in survival-linked textural biomarkers extracted from MBF and 0-MHz intercept parametric maps between treatment responders and non-responders, one week after the start of treatment. Whereas changes in mean values of mid-band fit and 0-MHz intercept only became statistically significant after four weeks of treatment. This study thus suggests for the first time that QUS spectroscopic methods in conjunction with textural analysis techniques can be used non-invasively to predict patient responses to clinical cancer therapies within days after treatment initiation. This work establishes a framework enabling acquisition of rapid and quantitative information to evaluate and predict responses of cancer patients to the treatments. Such a system can in the future facilitate customization of treatment for cancer patients on an individual basis.

## RESULTS

Characteristics of the patients who participated in this study, their tumor properties, and the treatments administrated have been summarized in Table 1A. The patients (n=20) had an average age of 45 years (SD=7.4, range: 33-57), and an average tumor size of 7.3 cm (SD=2.8, range: 3-13) with respect to the largest tumor dimension. Nineteen patients had invasive ductal carcinoma, one had metaplastic carcinoma, and eleven patients had tumors with positive estrogen and/or progesterone receptors (ER/PR+), whereas eight patients had a Her-2-Neu positive (HER2+) status. The majority of patients received combined anthracycline and taxane-based chemotherapy. The clinical/pathologic responses of patients to their neo-adjuvant chemotherapy are presented in Table 1B. Patients 1, 4, 5, 6, 7, 9, 12, 13, 14, 17, 19, and 20 had either a complete pathologic response, or had reductions of more than 50% in their tumor size along with detectable decreases in tumor cellularity, and were categorized as responders. In the case of patients 2, 15, and 16 a substantial reduction in physical size of mass was not detected, however residual tumor cellularity was very low and these patients were clinically/pathologically recognized as responders. Patients 3, 8, 10, 11, and 18 demonstrated progressive disease, or only slight changes in their tumor size during treatment along with high residual tumor cellularity, and were classified as non-responders.

Table 1Characteristics of participating patients (A), and their responses to treatment (B).

**Table:** A

No.	Age	Menop. status	Initial Tumor Dimensions (AP × ML ×SI) in cm	Histology	Grade	ER/PR	Her-2-neu	Neoadjuvant Treatment
1	55	N/A	5.4 × 5 × 2.3	ductal	N/A	-	+	FEC + paclitaxel, trastuzumab
2	53	N/A	7.4 × 7	ductal	2	+	-	Epirubicin, docetaxel
3	41	Pre	4	ductal	3	+	+	Docetaxel, carboplatin, trastuzumab
4	50	Pre	4 × 5	ductal	N/A	-	+	AC + docetaxel, trastuzumab
5	33	Pre	3 × 3	ductal	1	+	-	AC + paclitaxel
6	33	Pre	5.4 × 5 × 8	ductal	N/A	+	+	AC + docetaxel, paclitaxel, trastuzumab
7	48	Post	4.9 × 4.9 × 4.1 & 3.2 × 1.3 × 2.9	ductal	2	+	-	AC + docetaxel
8	36	Pre	4.4 × 3.9 × 5.8	ductal	2	+	-	AC + paclitaxel
9	40	Pre	4.4 × 3.4	ductal	3	-	-	AC + paclitaxel
10	38	Pre	7.5 × 4.9 × 9.2	ductal	2	+	-	AC + paclitaxel
11	53	N/A	8.4 × 9.4 × 12.7	meta plastic	3	-	-	AC + cisplatinum, gemcitabine platinum
12	50	Pre	13 × 11	ductal	3	-	-	AC + paclitaxel
13	49	Pre	7.1 × 5.5 × 8.9	ductal	3	-	+	Docetaxel, trastuzumab
14	40	Pre	3 × 2.4 × 3	ductal	3	+	+	AC + paclitaxel, trastuzumab
15	47	Pre	5.2 × 4 × 4	ductal	2	+	-	FEC + docetaxel
16	38	Pre	9 × 6.6 × 6	ductal	2	+	-	AC + paclitaxel
17	38	Pre	8 × 8	ductal	N/A	-	+	Dose-dense AC + paclitaxel, trastuzumab
18	47	Pre	8 × 10	ductal	2	+	-	Dose-dense AC + paclitaxel
19	57	Post	7.9 × 4.1 × 5.5	ductal	N/A	-	-	Dose-dense AC + paclitaxel
20	47	Pre	6.3 × 4.1 × 7.4	ductal	N/A	-	+	Dose-dense AC + paclitaxel, trastuzumab
AC: Adriamycin and Cytoxan; FEC: Fluorouracil (5FU), epirubicin and cyclophosphamide

**Table:** B

No.	Residual Tumor Dimensions (AP × ML × SI) in cm	Notes	Clinical/Pathologic Response
1	N/A	Complete pathologic response	Good
2	7 × 5 × 3	Carcinoma with mucinous features; very low cellularity	Good
3	2.7 × 2.5 × 2.4	Tumor cellularity remains very high	Poor
4	N/A	Complete pathologic response	Good
5	1.4	Good response	Good
6	N/A	Complete pathologic response	Good
7	1.4 × 1 × 1	Small volume of invasive tumor remaining	Good
8	11.4	Extensive residual disease	Poor
9	N/A	Complete pathologic response, with only fibrous tumor bed remaining	Good
10	6.5 × 3 × 7.3	Invasive ductal carcinoma remaining	Poor
11	All the breast	Residual tumor took up all the breast; no response	Poor
12	4	Good response	Good
13	2 × 1.5 × 1	Complete pathologic response, with only in situ disease remaining	Good
14	0.2 × 0.2	Complete pathologic response, with only in situ disease remaining	Good
15	6.5	Exceedingly low cellularity, thus overall tumor volume is also very low	Good
16	2.9 × 2 × 1.5 & 2 × 1.5 × 1	Tumor cellularity is low	Good
17	N/A	Complete pathologic response	Good
18	12.5 × 4.5 × 3.5	No definite response	Poor
19	N/A	No residual invasive carcinoma in the breast, only lymphovascular invasion remaining	Good
20	N/A	Complete pathologic response, only scattered in-situ component remaining	Good

**Figure 1:**
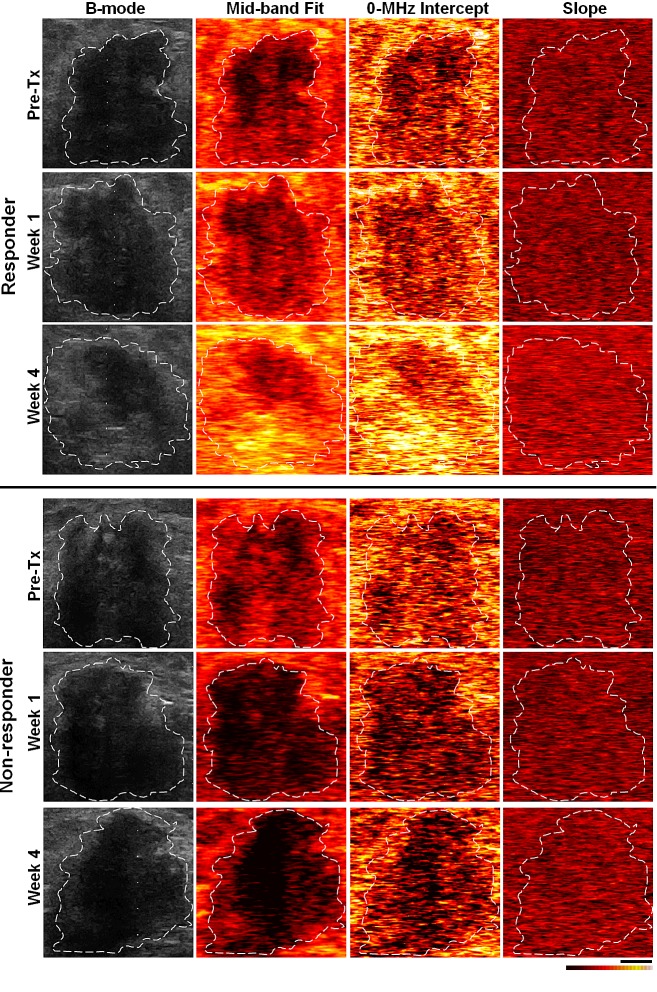
Representative ultrasound B-mode and spectral parametric images from a responding and a non-responding patient, acquired from the same nominal tumor regions (contoured by an oncologist), respectively, prior to the start of chemotherapy and after one and four weeks of treatment The scale bar is ~1 cm, and the color map represents a scale encompassing ~50 dBr for MBF and 0-MHz intercept, and ~20 dBr/MHz for the spectral slope.

**Figure 2:**
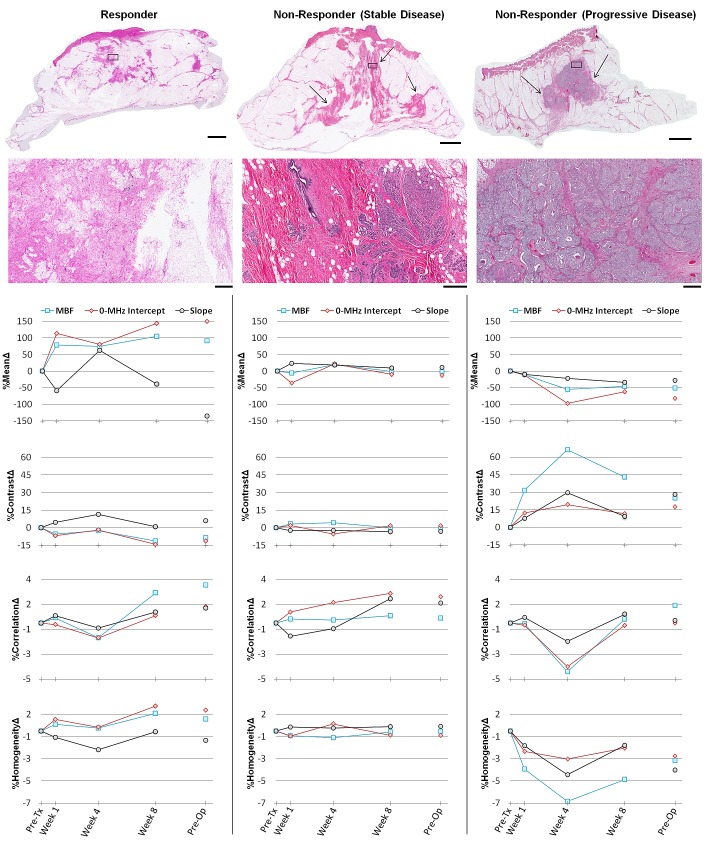
Representative data obtained from a responding patient (first column), and two non-responding patients with stable (second column) and progressive (third column) disease The first and second rows present light microscopy images of whole-mount histopathology slides obtained following modified radical mastectomy surgery, at low and high magnification, respectively. Areas of residual invasive carcinoma have been pointed out with arrows in low-magnification images. The corresponding areas of the high-magnification images have been marked with rectangles within the low-magnification images. The scale bars are ~1 cm and ~500 μm, in low and high-magnification images, respectively. The third to sixth rows demonstrate the results of ultrasound-based spectral and textural biomarkers measured for the same patients over the course of treatment. Data were measured prior to treatment onset, at weeks 1, 4 and 8 during treatment and preoperatively.

Representative ultrasound B-mode images, and spectral parametric maps corresponding to a responding and a non-responding patient, acquired from the same nominal regions of breast tumor, respectively, prior to the start of neo-adjuvant chemotherapy and after one and four weeks of treatment, are presented in Figure 1. An overall increase in the ultrasound spectral backscatter power was detectable within the tumor region of the responding patients, visualized as considerable changes in the MBF and 0-MHz intercept parametric images during the course of treatment. Particularly, increases of 3.7 ± 1.5 dBr and 6.8 ± 1.7 dBr for MBF, and 1.9 ± 0.9 dBr and 2.3 ± 0.5 dBr for 0-MHz intercept, were measured, on average, after one and four weeks of treatment for the responding patients. No such increase was observed in the case of non-responding patients, where changes of −2.1 ± 1.8 dBr and −4.9 ± 6.0 dBr for MBF, and −1.8 ± 1.4 dBr and −0.4 ± 1.6 dBr for 0-MHz intercept, were estimated, on average, after one and four weeks of treatment. Light microscopy images acquired from whole-mount histopathology sections of mastectomy specimens for a responding patient, and two non-responding patients with stable and progressive disease, respectively, are presented in Figure 2. In the cases of responding patients chemotherapy effect was clearly detectable within the tumor bed with minimal tumor cellularity remaining. In contrast, the whole-mount histopathology samples corresponding to the non-responding patients with stable or progressive disease indicated areas of residual disease with minimal chemotherapy effects in their mastectomy specimens, and a large intact residual mass, respectively. Similarly, the ultrasound-based spectral and textural biomarkers corresponding to these three patient types demonstrated different trends measurable over the course of treatment. Whereas the responding patients and the non-responding patients with progressive disease demonstrated opposite directions in the trend of a majority of the ultrasound-based biomarkers, the non-responding patients with stable disease exhibited minimal changes for these biomarkers (Figure 2).

**Figure 3:**
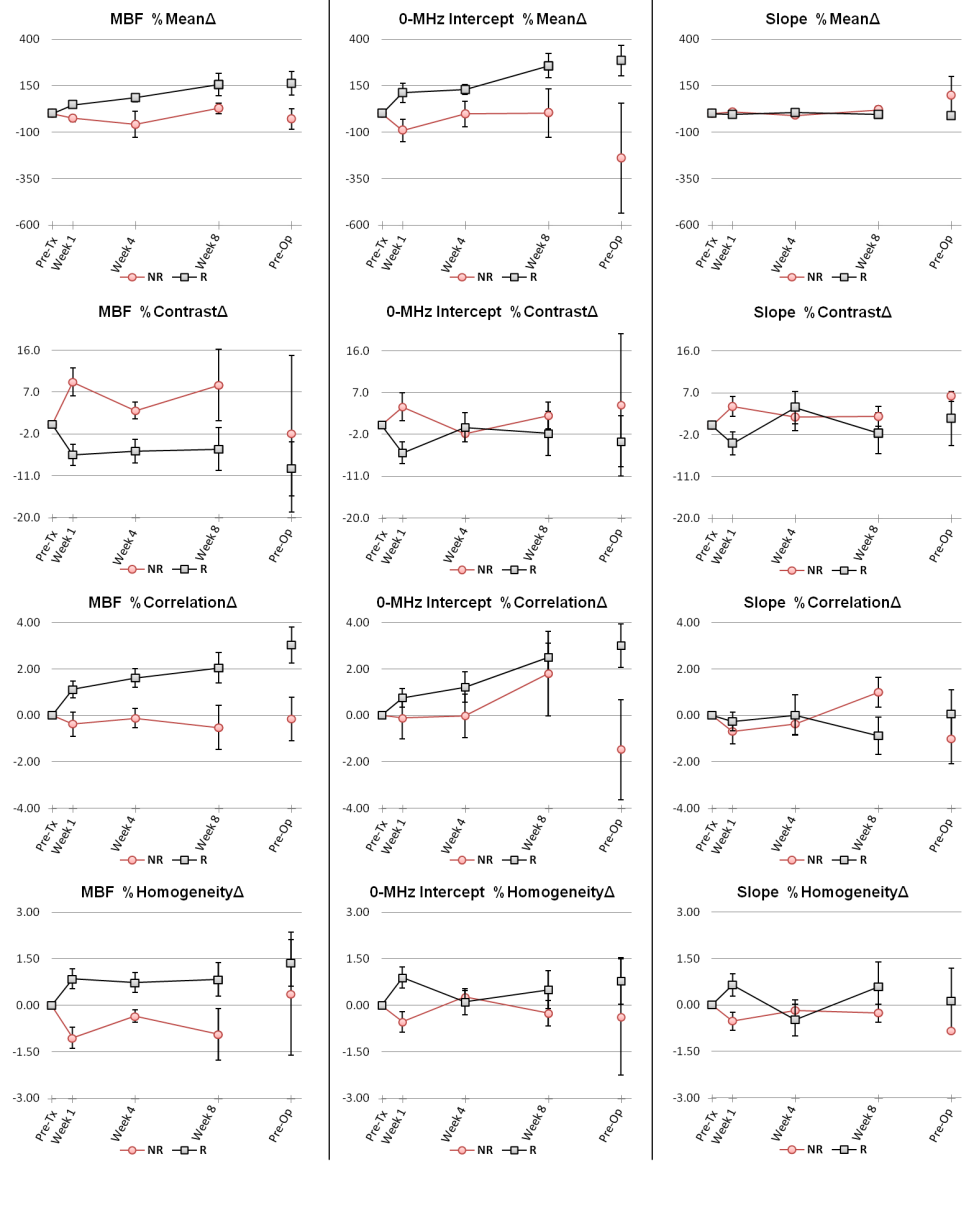
Average data obtained from treatment responding and non-responding patients during the course of treatment for the ultrasound-based spectral and textural biomarkers Data were measured prior to treatment initiation, at weeks 1, 4 and 8 during treatment and preoperatively. Red lines display results obtained from patients who were clinically/pathologically categorized as non-responders, whereas black lines display results obtained from responding patients. Error bars represent ± one standard error.

Average data obtained from responders and non-responding patients over the course of treatment is presented in Figure 3. Among the ultrasound-based parameters investigated, a large number of textural and spectral biomarkers exhibited a statistically significant difference between treatment responding and non-responding populations after one and four weeks of treatment. Particularly, statistically significant differences were observed after one week of treatment in MBF %ContrastΔ (p=0.002), MBF %CorrelationΔ (p=0.044), MBF %HomogeneityΔ (p=0.003), 0-MHz intercept %ContrastΔ (p=0.040), and 0-MHz intercept %HomogeneityΔ (p=0.035) between the two patient populations, whereas MBF %MeanΔ (p=0.054), 0-MHz intercept %MeanΔ (p=0.071), Slope %ContrastΔ (p=0.091), and Slope %HomogeneityΔ (p=0.090) approached the statistical significance. After four weeks of treatment, MBF %ContrastΔ (p=0.021), MBF %CorrelationΔ (p=0.028), MBF %HomogeneityΔ (p=0.021), MBF %MeanΔ (p=0.021), and 0-MHz intercept %MeanΔ (p=0.046) demonstrated statistically significant differences between the two populations. The mean of the slope parameter (slope %MeanΔ) remained almost invariant during the course of treatment within the two patient populations and was not demonstrated to be a statistically significant biomarker.

**Figure 4:**
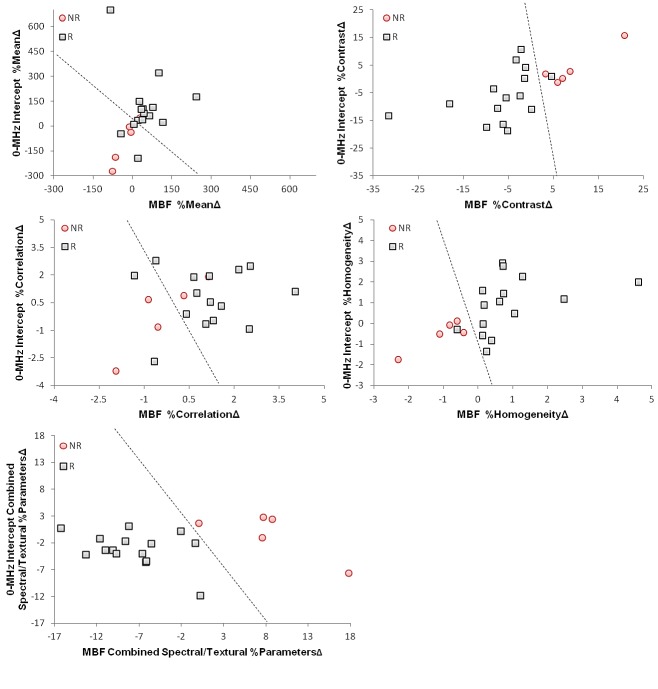
Scatter plots of the spectral, textural and hybrid biomarkers extracted from the MBF and 0-MHz intercept parametric images acquired from the patients one week after the start of chemotherapy Responding and non-responding patients have been classified in each feature plane via a linear discriminant analysis, where the determined border of classes has been demonstrated by a dashed line.

Results of discriminant analyses performed using the quantitative ultrasound-based biomarkers measured at week 1 of treatment are presented in Figures 4, and Table 2. Figure 4 illustrates the scatter plots of the patient data in the %MeanΔ, %ContrastΔ, %CorrelationΔ, and %HomogeneityΔ feature planes of MBF and 0-MHz intercept parameters, and in a combined feature plane of these spectral and textural biomarkers derived from their parametric images. In These plots, the determined borders of the treatment response classes have been shown by dashed lines. The plots suggest that the textural biomarkers, particularly %ContrastΔ and %HomogeneityΔ, provide considerably better separability between the two patient populations after one week of treatment, compared to the simple average-based values (%MeanΔ) of the spectral parameters. The results presented in Figure 4 also reflect a greater separability within a combined feature plane of the spectral and textural biomarkers derived from MBF and 0-MHz intercept parametric images. Table 2 summarizes the results of the ultrasound-based treatment response classification, in terms of sensitivities, specificities, and areas under the ROC curve obtained. In particular, the discriminant analyses resulted in sensitivities, specificities, and areas under the ROC curve of 100%, 93%, and 0.99 for MBF %ContrastΔ, 100%, 93%, and 0.97 for MBF %HomogeneityΔ, and 100%, 67%, and 0.80 for 0-MHz intercept %HomogeneityΔ after one week of treatment, whereas the MBF %MeanΔ demonstrated 80%, 80% and 0.84, and 0-MHz intercept %MeanΔ showed 80%, 80%, and 0.85 for sensitivity, specificity, and area under the ROC curve, respectively. The %ContrastΔ of MBF and 0-MHz intercept parameters in a combination, similar to %HomogeneityΔ, demonstrated a sensitivity of 100%, a specificity of 93%, and an area under the ROC curve of 0.99 for treatment response classification of the patients after one week of chemotherapy. A combination of the spectral and textural biomarkers extracted from parametric images of MBF and 0-MHz intercept (eight biomarkers in total), however, revealed the best results with sensitivity and specificity of 100% (p<0.001), and area under the ROC curve of 1.0.

**Table 2:** Results of early classification of patient ultimate responses to treatment based on quantitative ultrasound biomarkers at week 1

Parameters	LDA			ROC
	Sensitivity	Specificity	p-value	Area Under the Curve
MBF %MeanΔ	80%	80%	0.054	0.84
MBF %ContrastΔ	100%	93%	0.002	0.99
MBF %CorrelationΔ	80%	80%	0.044	0.81
MBF %HomogeneityΔ	100%	93%	0.005	0.97
0-MHz Intercept %MeanΔ	80%	80%	0.050	0.85
0-MHz Intercept %ContrastΔ	80%	67%	0.040	0.79
0-MHz Intercept %CorrelationΔ	40%	60%	0.318	0.65
0-MHz Intercept %HomogeneityΔ	100%	67%	0.035	0.80
Slope %MeanΔ	60%	47%	0.522	0.59
Slope %ContrastΔ	80%	60%	0.091	0.76
Slope %CorrelationΔ	60%	60%	0.592	0.61
Slope %HomogeneityΔ	80%	53%	0.090	0.76
MBF& 0-MHz Intercept %MeanΔ	80%	80%	0.032	0.89
MBF& 0-MHz Intercept %ContrastΔ	100%	93%	0.008	0.99
MBF& 0-MHz Intercept %CorrelationΔ	60%	87%	0.102	0.80
MBF& 0-MHz Intercept %HomogeneityΔ	100%	93%	0.008	0.99
MBF& 0-MHz Intercept Combined Spectral/Textural %ParametersΔ	100%	100%	< 0.001	1

**Figure 5:**
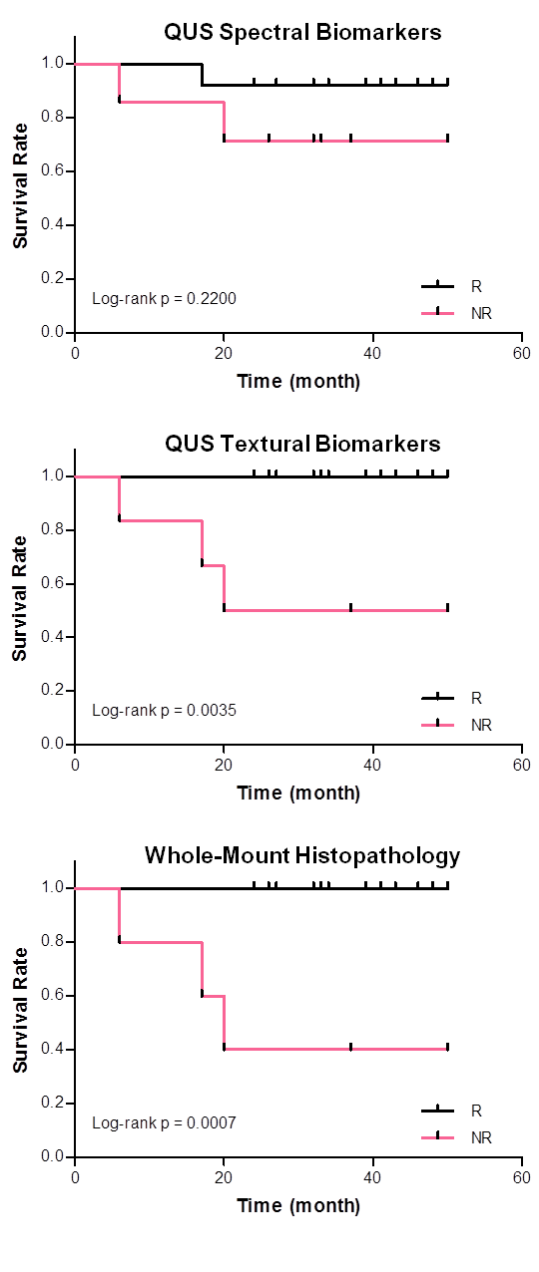
Kaplan-Meier survival curves of the responding and non-responding patient populations, determined based on quantitative ultrasound spectral biomarkers (first row) and textural biomarkers (second row) acquired one week after the start of chemotherapy The third row shows the survival curves determined based on whole-mount histopathology analysis on mastectomy specimens obtained after the surgery. Early survival analyses at week 1 based on the combined quantitative ultrasound spectral and textural biomarkers resulted in the same curves as obtained based on ultimate pathology of patients (third row).

Figure 5 demonstrates results of the recurrence-free survival analyses. The plots present the survival curves calculated for the responding and non-responding patient populations based on the QUS spectral (MBF and 0-MHz intercept %MeanΔ) and textural (MBF and 0-MHz intercept %ContratsΔ) biomarkers obtained one week after the start of chemotherapy, and those calculated months later based on ultimate clinical/pathologic responses of the patients. The survival curves obtained based on the QUS spectral biomarkers at week 1 of treatment did not show a statistically significant difference between the treatment outcomes of the two patient populations (p=0.220). However a statistically significant difference was observed between the survival curves obtained based on the QUS textural biomarkers at week 1 of the treatment (p=0.003). In addition, a statistically significant difference was found between the survival curves obtained months later based on the ultimate clinical/pathologic responses of the patients (p<0.001). Early survival analyses conducted at week 1 based on the combined QUS spectral and textural biomarkers in a hybrid profile also resulted in the same curves as obtained based on the ultimate clinical/pathologic responses.

## DISCUSSION

The results presented in this study indicate for the first time that alteration in the textural characteristics of QUS spectral parametric maps, complemented by changes in their mean values, can be non-invasively applied as early surrogates of clinical and pathologic treatment response exhibited by LABC patients to chemotherapy. Twenty women with LABC were monitored during the course of their neo-adjuvant chemotherapy and evaluated in terms of current clinical methods. The patients were also monitored with quantitative ultrasound-based spectral and textural parameters as non-invasive “biomarkers” of therapy response, measured for each patient over the course of treatment. The patients were consequently assessed after surgery with whole-mount histopathology analysis on mastectomy specimens. Obtained results demonstrate that the patients who were categorized ultimately as non-responders, according to the clinical and pathologic guidelines, exhibited considerably different trends for change in their corresponding ultrasound-based biomarkers over the course of treatment, compared to the clinical and pathologic responding patients. Statistically significant differences were exhibited between the two treatment response populations with respect to textural and spectral biomarkers, after one and four weeks of treatment, respectively.

Scatter plots of the patients' data in different feature planes of the quantitative ultrasound-based biomarkers demonstrated a very good separability of two treatment response populations at week 1 of chemotherapy. In this context, the separability provided by the ultrasound-based textural biomarkers after one week of treatment was better, compared to the one corresponding to the simple average-based spectral biomarkers. However, a hybrid profile of textural and spectral biomarkers resulted in the best separability between the two patient populations. In this case, the combination of spectral and textural biomarkers derived from the MBF and 0-MHz intercept parametric maps resulted in 100% sensitivity and specificity via a linear discriminant analysis performed for detecting non-responding patients. This implies that quantitative ultrasound-based spectral biomarkers can provide more information, when combined with the textural biomarkers, for treatment response monitoring, even at early stages after treatment. These QUS biomarkers, in a combination, may be applied for early prediction of ultimate treatment response in patients undergoing cancer-targeting therapies. Such an early prediction could be used to facilitate the decision of switching to a more effective therapy for treatment-refractory patients or even shifting to a salvage therapy, early on during a course of treatment.

Considering the levels of statistical significance of the textural and spectral biomarkers at week 1 and week 4 of treatment, the results observed in this study suggest that alterations in the textural properties of QUS spectral parametric maps become apparent at early stages of treatment, and will consequently result in more detectable changes in the mean values of these maps. This can be due to the fact that development of response in tumor cells is a gradual process which initially affects tissue micro-structures heterogeneously. In contrast to late effects that are more homogenous, the heterogeneous nature of the initial cell death progress and response development is expected to influence, at early stages, the textural properties of QUS spectral parametric images. Here, theses are demonstrated capable of characterizing tissue micro-structure effects. A gradual late accumulation of such heterogeneous alterations in the tissue micro-structures is anticipated to result in more detectable changes in the mean values of the QUS spectral parametric images at later stages [38], as confirmed in this study by a greater statistically significant difference between the two patient populations at week 4 of treatment. Previous pre-clinical studies on xenograft tumor models *in vivo* support the results observed in this study. In those studies, ultrasound-based textural parameters were found to be more sensitive to cell death, compared to mean values of the spectral parameters [41]. Textural parameters were also demonstrated to be capable of detecting changes in tissue micro-structures with a higher correlation to histological cell death, specially at early stages after chemotherapy exposure.

It was demonstrated in previous *in vitro* and *in vivo* investigations of ultrasound-based cell death detection that nuclear condensation and fragmentation in cell death can result in alterations in characteristics of ultrasonic backscatter signals, even at clinically-relevant conventional low frequencies [22,35,36,41,44]. This is consistent with observations in this study, in which such alterations were characterized by ultrasound-based spectral and textural parameters. Banihashmei *et al.* demonstrated that the cellular-based sub-resolution scatterers can affect ultrasound backscatter signal at low-frequency with cell death and evidence for the role of cell death related nuclear changes has been summarized there [23].

Results obtained in this study (Figure 3) demonstrated a lesser difference between responding and non-responding patients after eight weeks of treatment and months later prior to surgery. At the eighth week of treatment, the non-responders appear to show a late low level of response to therapy. In addition, a number of partial responders may have their tumor cells repopulated in partial regions exhibiting small levels of response. At the time of ultrasound data collection prior to surgery, the neo-adjuvant chemotherapy has been stopped for several weeks, and thus minimal cell death is expected. Also the complete pathologic responders, who have no residual tumor left in ultrasound scans, are not expected to show response and were excluded from the analysis at that time. Therefore, having less difference between the two patient populations can be expected at these times, since changes in the quantitative ultrasound-based biomarkers are expected to show the development of response for each patient.

Attenuation was accounted for in this study by a sliding window normalization process with data normalized using a tissue-mimicking phantom data, acquired under identical scan settings. In addition, the 0-MHz intercept, sensitive to the concentration of acoustic scatterers was derived, with parametric maps generated for each scan, as it is believed theoretically to be free of attenuation effects [40].

Previous studies have investigated the application of other functional imaging modalities for cancer treatment response monitoring. Examples include the modalities based on magnetic resonance imaging (MRI), positron emission tomography (PET), diffuse optical imaging (DOI), and elastography [25,26,31-33,43]. Unlike the methods based on MRI and PET modalities, the ultrasound-based biomarkers investigated in this study rely on intrinsic contrast alterations arising from changes in the acoustical characteristics of cancer cells when they die, and hence the method does not require the injection of any exogenous contrast agent. Elastography techniques have recently been reported useful for distinguishing between treatment responding and non-responding patients at the fourth week of chemotherapy, but not as early as one week [33]. In methods based on diffuse optical imaging, the lower resolution available may cause uncertainties for determining tumor boundaries, specially in the case of smaller tumors. Ultrasound is a portable and high resolution imaging modality that has the advantages of low cost and short imaging time, and can access tumor location not easily visualized with that modality. Genetic approaches have also been investigated recently for cancer therapy response monitoring [34]. Compared to these approaches, QUS biomarkers can provide a non-invasive insight of treatment response, needless of time-consuming analyses for quantification of circulating tumor DNA and gene sequencing.

In the study here, week 1 ultrasound biomarkers already indicated links to differential patient outcomes in terms of progression-free survival. At week 1, albeit in a small patient population, the combined ultrasound-based biomarkers in a hybrid profile resulted in outcomes which matched those based on ultimate pathology of patients, available months later, after their surgery.

In conclusion, this study demonstrates for the first time that early alterations in textural characteristics of QUS spectral parametric maps, complemented by changes in their mean values, can be clinically applied to predict ultimate clinical and pathologic responses of breast cancer patients to chemotherapy. Results indicate that treatment-refractory patients demonstrated different trends in measured ultrasound-based biomarkers over the course of treatment, compared to clinical and pathologic responding patients, and with statistical significance after one week and at the fourth week of treatment. The proposed biomarkers were also found to have a very good sensitivity and specificity to distinguish patients with poor ultimate response to the therapy, early-on following treatment initiation, and even with more accuracy when combined into a hybrid ultrasound-response profile. The promising results presented in this study suggest that these quantitative ultrasound-based biomarkers, as early survival-linked surrogates of ultimate treatment response to cancer-targeting therapies, may be applied to facilitate switching an inefficient treatment regimen for a particular refractory patient to a more effective one, or even undertaking a salvage treatment, early on after the therapy initiation [8].

## MATERIAL AND METHODS

### Study Protocol

This study was conducted in accordance with institutional research ethics approval from Sunnybrook Health Sciences Centre. The study was open to all women with LABC aged 18 to 85. Eligible patients were recruited for the study after obtaining written informed consent. Prior to therapy, all patients underwent a core needle biopsy to confirm a cancer diagnosis, where information regarding histological subtype and hormone receptor status of tumor were recorded. Pre-treatment magnetic resonance (MR) images of the breast were acquired for each patient in order to determine initial tumor size and to perform a metastatic workup as necessary as part of the institutional standard of clinical care for such patients. Patients were followed clinically by oncologists who remained blinded to the study results. Physical examination was conducted with each cycle of chemotherapy and size and stiffness of tumor was assessed by clinicians. Post-treatment MRI scans of the breast were also acquired immediately before patient surgery to measure residual tumor size. Patients were also followed clinically up to 50 months after their treatment and their clinical data were recorded for a recurrence-free survival study.

Following surgery, patient mastectomy specimens were mounted on whole-mount [45] 5”×7” pathology slides and were consequently stained with haematoxylin and eosin (H&E). The pathology slides were digitized using a confocal scanner (TISSUEscope™, Huron Technologies, Waterloo, ON) at 2 micron resolution. All cases were examined by the same pathologist, who provided information regarding tumor grade, residual size, extent of cellularity, and tumor response. Patient responses were categorized following standard guidelines based upon changes in overall tumor volume in addition to the residual tumor cellularity [27,46,47]. Patients were considered as responders if there was a decrease in tumor size of 50% or more, and included patients which were deemed to have a complete pathologic response to treatment (no residual invasive carcinoma) [27,46,47]. Conversely, patients were deemed to be non-responders if there was less than a 50% decrease in tumor size and included patients with progressive disease in which the tumor volume increased despite treatment [27,46,47]. In cases where the tumor cellularity was very low (overall volume of viable tumor cells), the patient was considered as a responder as well, even if the diminishment in the physical tumor size was less than 50%.

### Ultrasound Data Collection

All the ultrasound data in this study were collected by the same sonographer following standardized protocols for data acquisition. Ultrasound data was acquired with patients lying supine with their arms above their heads. Ultrasound data was acquired at 5 times during course of treatment for each patient. The first scan was acquired immediately prior to the start of chemotherapy which was used as a baseline of comparison for subsequent scans. The following three scans were acquired during the first, fourth and eighth week of treatment, with a fifth scan acquired within one week prior to the modified radical mastectomy surgery. The mastectomy surgery was typically carried out four to six weeks after the course of chemotherapy was completed.

Conventional B-mode images and ultrasound radiofrequency (RF) data were acquired using a Sonix RP, (Ultrasonix Vancouver, Canada) system utilizing a L14-5/60 transducer with a transmit frequency of 10 MHz, resulting in a frequency bandwidth with a centre frequency of ~7 MHz. The ultrasound RF data were digitized with a sampling frequency of 40 MHz. The transducer focus was set at varying depths depending on individual patient circumstances. Scan focal depths remained consistent for individual patients throughout the study. Breast regions selected for ultrasound scanning were directed by an oncologist, who determined acquisition scan planes via physical examination of the patient. Data was acquired in a single continuous sweep over the entire tumor volume in order to provide context regarding changes in localization and dimensionality of the tumor across visits. Scans of individual tumor regions were also acquired at approximately 1 cm increments across the whole tumor volume.

### Ultrasound Data Analysis

Analysis of ultrasound RF data was carried out using quantitative ultrasound spectroscopy, followed by textural analysis on parametric images of the spectral biomarkers obtained. Quantitative ultrasound spectroscopy was performed using linear regression analysis of the normalized power spectrum [22,23,35,36,39,40], applying a sliding window analysis for generating parametric images. Ultrasound data was analyzed across all acquired planes through the scan volume which included identifiable tumor regions, and parametric images were generated for each cross-sectional plane (described further below). Analysis parameters were reported from data within a region of interest (ROI) located at the tumor central area which was consistently positioned at the transducer focal depth, typically accounting for approximately two third of the tumor area in the plane. Obtained values of parameters for each scan were reported as tumor volumetric averages (detailed below).

The power spectrum was calculated using a Fourier transform of the raw RF signal for each scan line through the whole field of view of the ultrasound data. In order to remove the effects of system transfer functions, transducer beam-forming, and diffraction artefacts, in addition to acting as a mechanism of depth related attenuation correction, data were normalized using a sliding window analysis with the power spectrum obtained from a glass-bead-embedded agar-gel phantom model [48], with properties similar to those of breast tissue (modified from [49]). Phantom data was acquired for each setting used during patient data acquisition, including variations in image gain and focal depth. Each sliding window was normalized separately to a reference curve obtained from the same region of the phantom, with equivalent location and size. This was carried out in order to more accurately account for the effects of attenuation and beam diffraction across the region of interest especially in larger tumors. Linear regression analysis was performed on the normalized power spectrum within a −6 dB window from the transducer centre frequency determined from a calibration pulse, to generate a best-fit line. Parameters subsequently reported included the mid-band fit, the spectral slope, and the corresponding 0-MHz intercept [23,35,39,40], as ultrasound-based spectral biomarkers of treatment response. The parametric images were generated using the sliding window analysis on a pixel by pixel basis, with the parameters calculated for each window and assigned to its centre. The size of the sliding window was selected to cover approximately 10 wavelengths in order to obtain more reliable spectral parameters which are independent of window length [50].

In addition to the mean values of these spectral biomarkers determined by averaging on the generated parametric images, textural properties of each parametric map were also characterized to derive ultrasound-based textural biomarkers of heterogeneous therapy response. Textural analysis was performed based on a gray-level co-occurrence matrix (GLCM) [51]. Such a matrix represents the angular relationship between neighboring pixels as well as the distance between them [52], comprising high-order textural information about patterns of neighboring pixels in an image. Symmetric GLCMs were constructed considering each pixel's neighbors located at different distances and directions, *i.e.*, at angles of 0° (180°), 45° (225°), 90° (270°), and 135° (315°). Textural parameters (contrast, correlation, and homogeneity) were extracted from the corresponding GLCMs of each spectral parametric image, and were subsequently averaged [51]. Among the textural parameters determined, contrast represents a measure of difference between the lowest and highest intensities in a set of pixels, correlation quantifies the intensity correlation between pixel pairs, and homogeneity measures the incidence of pixel pairs of different intensities. All the mean values and the textural parameters were determined for each of the scan planes collected per patient visit, and subsequently averaged across the tumor volume.

Comparison of each patient's data during the course of treatment was conducted using their corresponding data acquired prior to the treatment initiation, as the baseline. The values of each quantitative parameter for clinically and pathologically determined responders and non-responders were compared independently for each time. Normality violations for each parameter were examined using a one-sample Kolmogorov-Smirnov test (PASW Statistics 18, SPSS Inc., Chicago, IL). Depending on whether the changes were normally distributed or not, statistical analyses using either a t-test or a two-sample Kolmogorov-Smirnov test (two-sided, 95% confidence) were carried out, respectively, to assess if responding and non-responding patient populations exhibit statistically significant differences in changes measured for the quantitative ultrasound-based biomarkers during the course of treatment. Linear discriminant analyses were used to determine which quantitative parameter better discriminated between responders and non-responders. Sensitivity and specificity were calculated in addition to receiver operating characteristics (ROC), in order to measure the performance of the quantitative ultrasound-based treatment response classification method compared to clinically and pathologically determined responses. Survival analyses were performed to generate recurrence-free Kaplan-Meier survival curves for responding and non-responding patient populations, determined based on ultrasound-based biomarkers, and based on ultimate clinical/pathologic responses. Survival curves obtained for the two patient populations were compared using a log-rank test to evaluate if they demonstrate statistically significant differences between the treatment outcomes.

## References

[1] Huang E, McNeese MD, Strom EA, Perkins GH, Katz A, Hortobagyi GN, Valero V, Kuerer HM, Singletary SE, Hunt KK, Buzdar AU, Buchholz TA (2002). Locoregional treatment outcomes for inoperable anthracycline-resistant breast cancer. Int J Radiat Oncol Biol Phys.

[2] Leibovici D, Spiess PE, Heller L, Rodriguez-Bigas M, Chang G, Pisters LL (2008). Salvage surgery for locally recurrent prostate cancer after radiation therapy: tricks of the trade. Urol Oncol.

[3] Wistuba II, Gelovani JG, Jacoby JJ, Davis SE, Herbst RS (2011). Methodological and practical challenges for personalized cancer therapies. Nat Rev Clin Oncol.

[4] Juweid ME, Cheson BD (2006). Positron-emission tomography and assessment of cancer therapy. N Engl J Med.

[5] Sikora K (2005). Personalized cancer therapy. Per Med.

[6] McDermott U, Settleman J (2009). Personalized cancer therapy with selective kinase inhibitors: an emerging paradigm in medical oncology. J Clin Oncol.

[7] Dowsett M, Dunbier AK (2008). Emerging biomarkers and new understanding of traditional markers in personalized therapy for breast cancer. Clin Cancer Res.

[8] Von Minckwitz G, Blohmer JU, Costa SD, Denkert C, Eidtmann H, Eiermann W, Gerber B, Hanusch C, Hilfrich J, Huober J, Jackisch C, Kaufmann M, Kümmel S, Paepke S, Schneeweiss A, Untch M, Zahm DM, Mehta K, Loibl S (2013). Response-guided neoadjuvant chemotherapy for breast cancer. J Clin Oncol.

[9] American Cancer Society (2012). Cancer facts and figures 2012. Atlanta, GA, USA.

[10] Mankoff DA, Dunnwald LK, Gralow JR, Ellis GK, Drucker MJ, Livingston RB (1999). Monitoring the response of patients with locally advanced breast carcinoma to neoadjuvant chemotherapy using [technetium 99m]-sestamibi scintimammography. Cancer.

[11] Giordano SH (2003). Update on locally advanced breast cancer. Oncologist.

[12] Esteva FJ, Hortobagyi GN (1999). Locally advanced breast cancer. Hematol Oncol Clin North Am.

[13] Hortobagyi GN (1990). Comprehensive management of locally advanced breast cancer. Cancer.

[14] De Lena M, Varini M, Zucali R, Rovini D, Viganotti G, Valagussa P, Veronesi U, Bonadonna G (1981). Multimodal treatment for locally advanced breast cancer. Result of chemotherapy-radiotherapy versus chemotherapy-surgery. Cancer Clin Trials.

[15] Smith IC, Heys SD, Hutcheon AW, Miller ID, Payne S, Gilbert FJ, Ah-See AK, Eremin O, Walker LG, Sarkar TK, Eggleton SP, Ogston KN (2002). Neoadjuvant chemotherapy in breast cancer: significantly enhanced response with docetaxel. J Clin Oncol.

[16] Chollet P, Charrier S, Brain E, Curé H, van Praagh I, Feillel V, de Latour M, Dauplat J, Misset JL, Ferrière JP (1997). Clinical and pathological response to primary chemotherapy in operable breast cancer. Eur J Cancer.

[17] Yeh E, Slanetz P, Kopans DB, Rafferty E, Georgian-Smith D, Moy L, Halpern E, Moore R, Kuter I, Taghian A (2005). Prospective comparison of mammography, sonography, and MRI in patients undergoing neoadjuvant chemotherapy for palpable breast cancer. Am J Roentgenol.

[18] Mueller MM, Fusenig NE (2004). Friends or foes - bipolar effects of the tumour stroma in cancer. Nat Rev Cancer.

[19] Schedin P, O'Brien J, Rudolph M, Stein T, Borges V (2007). Microenvironment of the involuting mammary gland mediates mammary cancer progression. J Mammary Gland Biol Neoplasia.

[20] Varghese T, Zagzebski JA, Lee FT (2002). Elastographic imaging of thermal lesions in the liver in vivo following radiofrequency ablation: preliminary results. Ultrasound Med Biol.

[21] Kolokythas O, Gauthier T, Fernandez AT, Xie H, Timm BA, Cuevas C, Dighe MK, Mitsumori LM, Bruce MF, Herzka DA, Goswami GK, Andrews RT, Oas KM, Dubinsky TJ, Warren BH (2008). Ultrasound-based elastography: a novel approach to assess radio frequency ablation of liver masses performed with expandable ablation probes: a feasibility study. J ultrasound Med.

[22] Czarnota GJ, Kolios MC, Abraham J, Portnoy M, Ottensmeyer FP, Hunt JW, Sherar MD (1999). Ultrasound imaging of apoptosis: high-resolution non-invasive monitoring of programmed cell death in vitro, in situ and in vivo. Br J Cancer.

[23] Banihashemi B, Vlad R, Debeljevic B, Giles A, Kolios MC, Czarnota GJ (2008). Ultrasound imaging of apoptosis in tumor response: novel preclinical monitoring of photodynamic therapy effects. Cancer Res.

[24] Tromberg BJ, Shah N, Lanning R, Cerussi A, Espinoza J, Pham T, Svaasand L, Butler J (2000). Non-invasive in vivo characterization of breast tumors using photon migration spectroscopy. Neoplasia.

[25] Brindle K (2008). New approaches for imaging tumour responses to treatment. Nat Rev Cancer.

[26] Sadeghi-Naini A, Falou O, Hudson JM, Bailey C, Burns PN, Yaffe MJ, Stanisz GJ, Kolios MC, Czarnota GJ (2012). Imaging innovations for cancer therapy response monitoring. Imaging Med.

[27] Therasse P, Arbuck SG, Eisenhauer EA, Wanders J, Kaplan RS, Rubinstein L, Verweij J, Van Glabbeke M, van Oosterom AT, Christian MC, Gwyther SG (2000). New guidelines to evaluate the response to treatment in solid tumors. J Natl Cancer Inst.

[28] Machida N, Yoshino T, Boku N, Hironaka S, Onozawa Y, Fukutomi A, Yamazaki K, Yasui H, Taku K, Asaka M (2008). Impact of baseline sum of longest diameter in target lesions by RECIST on survival of patients with metastatic colorectal cancer. Jpn J Clin Oncol.

[29] Witney TH, Brindle KM (2010). Imaging tumour cell metabolism using hyperpolarized 13C magnetic resonance spectroscopy. Biochem Soc Trans.

[30] Witney TH, Kettunen MI, Hu D, Gallagher FA, Bohndiek SE, Napolitano R, Brindle KM (2010). Detecting treatment response in a model of human breast adenocarcinoma using hyperpolarised [1-13C]pyruvate and [1,4-13C^2^]fumarate. Br J Cancer.

[31] Soliman H, Gunasekara A, Rycroft M, Zubovits J, Dent R, Spayne J, Yaffe MJ, Czarnota GJ (2010). Functional imaging using diffuse optical spectroscopy of neoadjuvant chemotherapy response in women with locally advanced breast cancer. Clin Cancer Res.

[32] Falou O, Soliman H, Sadeghi-Naini A, Iradji S, Lemon-Wong S, Zubovits J, Spayne J, Dent R, Trudeau M, Boileau JF, Wright FC, Yaffe MJ, Czarnota GJ (2012). Diffuse optical spectroscopy evaluation of treatment response in women with locally advanced breast cancer receiving neoadjuvant chemotherapy. Transl Oncol.

[33] Falou O, Sadeghi-Naini A, Prematilake S, Sofroni E, Papanicolau N, Iradji S, Jahedmotlagh Z, Lemon-Wong S, Pignol J-P, Rakovitch E, Zubovits J, Spayne J, Dent R, Trudeau M, Boileau JF, Wright FC, Yaffe MJ, Czarnota GJ (2013). Evaluation of neoadjuvant chemotherapy response in women with locally advanced breast cancer using ultrasound elastography. Transl Oncol.

[34] Dawson S-J, Tsui DWY, Murtaza M, Biggs H, Rueda OM, Chin S-F, Dunning MJ, Gale D, Forshew T, Mahler-Araujo B, Rajan S, Humphray S, Becq J, Halsall D, Wallis M, Bentley D, Caldas C, Rosenfeld N (2013). Analysis of circulating tumor DNA to monitor metastatic breast cancer. N Engl J Med.

[35] Sadeghi-Naini A, Papanicolau N, Falou O, Tadayyon H, Lee J, Zubovits J, Sadeghian A, Karshafian R, Al-Mahrouki A, Giles A, Kolios MC, Czarnota GJ (2013). Low-frequency quantitative ultrasound imaging of cell death in vivo. Med Phys.

[36] Vlad RM, Brand S, Giles A, Kolios MC, Czarnota GJ (2009). Quantitative ultrasound characterization of responses to radiotherapy in cancer mouse models. Clin cancer Res.

[37] Czarnota GJ, Karshafian R, Burns PN, Wong S, Al Mahrouki A, Lee JW, Caissie A, Tran W, Kim C, Furukawa M, Wong E, Giles A (2012). Tumor radiation response enhancement by acoustical stimulation of the vasculature. Proc Natl Acad Sci U S A.

[38] Sadeghi-Naini A, Papanicolau N, Falou O, Zubovits J, Dent R, Verma S, Trudeau ME, Boileau JF, Spayne J, Iradji S, Sofroni E, Lee J, Lemon-Wong S, Yaffe MJ, Kolios MC, Czarnota GJ (2013). Quantitative Ultrasound Evaluation of Tumour Cell Death Response in Locally Advanced Breast Cancer Patients Receiving Chemotherapy. Clin cancer Res.

[39] Lizzi FL, Ostromogilsky M, Feleppa EJ, Rorke MC, Yaremko MM (1987). Relationship of ultrasonic spectral parameters to features of tissue microstructure. IEEE Trans Ultrason Ferroelectr Freq Control.

[40] Lizzi FL, Astor M, Liu T, Deng C, Coleman DJ, Silverman RH (1997). Ultrasonic spectrum analysis for tissue assays and therapy evaluation. Int J Imaging Syst Technol.

[41] Sadeghi-Naini A, Falou O, Tadayyon H, Al-Mahrouki A, Tran W, Papanicolau N, Kolios MC, Czarnota GJ (2013). Conventional frequency ultrasonic biomarkers of cancer treatment response in vivo. Transl Oncol.

[42] Sadeghi-Naini A, Falou O, Czarnota GJ (2013). Characterizing tumour heterogeneous response to chemotherapy using low-frequency ultrasonic spectroscopy. Proceedings of Meetings on Acoustics – 21st International Congress on Acoustics (ICA). Montreal, QC, Canada.

[43] Larkin TJ, Canuto HC, Kettunen MI, Booth TC, Hu D-E, Krishnan AS, Bohndiek SE, Neves A a, McLachlan C, Hobson MP, Brindle KM (2014). Analysis of image heterogeneity using 2D Minkowski functionals detects tumor responses to treatment. Magn Reson Med.

[44] Czarnota GJ, Kolios MC, Vaziri H, Benchimol S, Ottensmeyer FP, Sherar MD, Hunt JW (1997). Ultrasonic biomicroscopy of viable, dead and apoptotic cells. Ultrasound Med Biol.

[45] Clarke GM, Eidt S, Sun L, Mawdsley G, Zubovits JT, Yaffe MJ (2007). Whole-specimen histopathology: a method to produce whole-mount breast serial sections for 3-D digital histopathology imaging. Histopathology.

[46] Roblyer D, Ueda S, Cerussi A, Tanamai W, Durkin A, Mehta R, Hsiang D, Butler JA, McLaren C, Chen W-P, Tromberg B (2011). Optical imaging of breast cancer oxyhemoglobin flare correlates with neoadjuvant chemotherapy response one day after starting treatment. Proc Natl Acad Sci U S A.

[47] Cerussi A, Hsiang D, Shah N, Mehta R, Durkin A, Butler J, Tromberg BJ (2007). Predicting response to breast cancer neoadjuvant chemotherapy using diffuse optical spectroscopy. Proc Natl Acad Sci U S A.

[48] Yao LX, Zagzebski JA, Madsen EL (1990). Backscatter coefficient measurements using a reference phantom to extract depth-dependent instrumentation factors. Ultrason Imaging.

[49] Dong F, Madsen EL, MacDonald MC, Zagzebski JA (1999). Nonlinearity parameter for tissue-mimicking materials. Ultrasound Med Biol.

[50] Topp KA, Zachary JF, O'Brien WD (2001). Quantifying B-mode images of in vivo rat mammary tumors by the frequency dependence of backscatter. J ultrasound Med.

[51] Haralick RM, Shanmugam K, Dinstein I (1973). Textural Features for Image Classification. IEEE Trans Syst Man Cybern.

[52] Liao Y-Y, Tsui P-H, Li C-H, Chang K-J, Kuo W-H, Chang C-C, Yeh C-K (2011). Classification of scattering media within benign and malignant breast tumors based on ultrasound texture-feature-based and Nakagami-parameter images. Med Phys.

